# Multiple Desmoplastic Small Round Cell Tumor in the Intestine: A Case Report

**DOI:** 10.70352/scrj.cr.24-0135

**Published:** 2025-02-28

**Authors:** Naoto Tsujimura, Mitsuyoshi Tei, Daisuke Umeda, Koki Ishimaru, Shoko Minamiura, Takehiro Yamamoto, Soichiro Mori, Kentaro Nishida, Yukihiro Yoshikawa, Masatoshi Nomura, Koki Tamai, Takuya Hamakawa, Daisuke Takiuchi, Hironao Yasuoka, Masanori Tsujie, Yusuke Akamaru

**Affiliations:** 1 Department of Gastroenterological Surgery, Osaka Rosai Hospital, Sakai, Osaka, Japan; 2 Department of Diagnostic Pathology, Osaka Rosai Hospital, Sakai, Osaka, Japan

**Keywords:** desmoplastic small round cell tumor, multiple tumor, intestine, prognosis

## Abstract

**INTRODUCTION:**

Desmoplastic small round cell tumor (DSRCT) is a highly malignant sarcoma and an extremely rare tumor, predominantly found in the abdominal and pelvic regions. Here, we report the case of a patient who underwent surgical treatment for multiple desmoplastic round cell tumor in the intestine.

**CASE PRESENTATION:**

A 38-year-old male patient visited our hospital after a health check revealed positive occult blood in his stool and a colonoscopy revealed tumors in descending colon and sigmoid colon. Biopsy results revealed poorly differentiated adenocarcinoma. Chest and abdominal enhanced computed tomography revealed 3 tumors from descending colon to sigmoid colon and numerous peritoneal disseminations. Based on these findings, we diagnosed multiple colon cancers and performed a laparoscopic left hemicolectomy. Hematoxylin–Eosin (H&E) staining showed that in all tumors, atypical cells with large and small swollen nuclei formed irregular solid nests of various sizes against a background of extensive desmoplastic or myxomatous stroma. Immunohistochemistry showed that tumor cells were AE1/3 (+), S-100 (–), Desmin (–), WT1 (–). Genetic analysis detected the Ewing’s sarcoma and Wilms tumor fusion gene at another inspection agency. Histopathological examination identified desmoplastic small round cell tumor. The patient was discharged on the 19th postoperative day without postoperative complications. He will undergo chemotherapy at another hospital.

**CONCLUSIONS:**

We experienced a very rare case of DSRCT. DSRCT is a fatal disease that primarily affects adolescent and young adult males. Currently, there is no proven treatment. More case reports are essential to improve management of this disease.

## Abbreviations

AE1/3
: Cytokeratin-multi (AE1/3)
ALP
: alkaline phosphatase
ALT
: alanine aminotransferase
AR-ASO
: androgen receptor-directed antisense oligonucleotides
AST
: aspartate aminotransferase
CA125
: carbohydrate antigen 125
CEA
: carcinoembryonic antigen
CK
: Cytokeratin
CNA
: copy number alterations
CRP
: C-reactive protein
CRS
: cytoreductive surgery
CT
: computed tomography
c-MYC
: Myc proto-oncogene protein
DDR
: DNA damage repair
DST
: double stapling technique
DSRCT
: desmoplastic small round cell tumor
EGFR
: epidermal growth factor receptor
EMA
: epithelial membrane antigen
EMT
: epithelial–mesenchymal transition
EWS
: Ewing sarcoma
EWSR1
: Ewing’s sarcoma breakpoint region1
FGFR
: fibroblast growth factor receptor
H&E
: hematoxylin-Eosin
HGB
: hemoglobin
HIPEC
: hyperthermic intraperitoneal chemotherapy
IGF-1
: insulin-like growth factor-1
LDH
: lactate dehydrogenase
LYM
: lymphocyte
LYM-R
: lymphocyte rate
MErT
: mesenchymal–epithelial reverse transition
MRI
: magnetic resonance imaging
NEU
: neutrophil
NEU-R
: neutrophil rate
NSE
: Neuron-specific enolase
PDGF
: platelet-derived growth factor
PET
: positron emission tomography
PLT
: platelet
RTK
: receptor tyrosine kinase
SD
: Sigmoid-Descending colon
SMT
: submucosal tumor
T2WI
: T2 weighted image
T-BIL
: total bilirubin
WBC
: white blood cell
WT1
: Wilms tumor

## INTRODUCTION

Desmoplastic small round cell tumor (DSRCT) is a highly malignant sarcoma and an extremely rare tumor predominantly found in the abdominal and pelvic regions.^1–4)^ Initially described by Gerald and Rosai in 1989, it was officially named in 1991.^5–7)^ DSRCT accounts for <1% of all soft tissue sarcomas and is more prevalent in men, with a peak incidence in the 20s. DSRCT is characterized by small round cells of various sizes within a densely proliferating fibrous stromal matrix.^8)^ The diagnosis is confirmed by detection of the specific chromosomal translocation t (11;22) (p13;q12). This translocation results in an active fusion protein involving the Ewing sarcoma (EWS) and Wilms tumor (WT1) genes and is pathognomonic for the diagnosis.^9)^ DSRCT can metastasize early and recur rapidly despite treatment. Thus, the prognosis is very poor. Despite multimodal treatment based on chemotherapy, surgery, and radiation therapy, durable remission remains rare. We report a case of DSRCT that is a highly malignant sarcoma and extremely rare.

## CASE PRESENTATION

A 38-year-old male patient visited our hospital in June 2024. A health check revealed positive occult blood in his stool, and a colonoscopy revealed tumors in the descending colon and sigmoid colon. Physical examination revealed that the patient was in good general condition, had no abdominal pain, and no palpable masses. Blood test findings revealed a white blood cell count of 5470/μL, hemoglobin of 13.8 g/dL, C-reactive protein (CRP) of 0.2 mg/dL, aspartate aminotransferase (AST) of 32 U/L, and alanine aminotransferase (ALT) of 54 U/L. These findings indicated mild liver dysfunction. Tumor marker assessment revealed carcinoembryonic antigen (CEA) of 2.6 ng/mL and CA19-9 of 6 U/mL, both within the normal range (**Table 1**).

**Table 1:** Partial laboratory test results

Item	Results	Units
Routine blood test		
WBC	5.47	10^3^/μL
NEU	3.047	10^3^/μL
NEU-R	55.7	%
LYM	1.849	10^3^/μL
LYM-R	33.8	%
HGB	13.8	g/dL
PLT	22.6	10^4^/μL
Biochemistry test		
ALT	54	U/L
AST	32	U/L
T-BIL	0.5	mg/dL
LDH	129	U/L
ALP	104	U/L
Urea	10	mg/dL
Crea	0.8	mg/dL
Serum tumor markers		
CEA	2.6	ng/mL
CA19-9	6	U/mL

ALP, alkaline phosphatase; ALT, alanine aminotransferase; AST, aspartate aminotransferase; CA19-9, carbohydrate antigen 19-9; CEA, carcinoembryonic antigen; HGB, hemoglobin; LDH, lactate dehydrogenase; LYM, lymphocyte; LYM-R, lymphocyte rate; NEU, neutrophil; NEU-R, neutrophil rate; PLT, platelet; T-BIL, total bilirubin; WBC, white blood cell

**Fig. 1:**
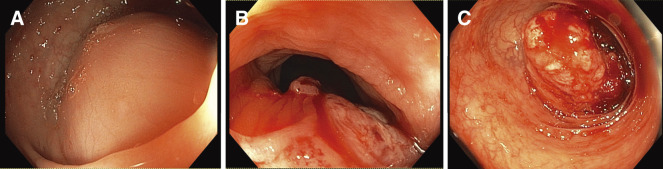
Preoperative colonoscopy. (**A**) The SMT-like tumor was located in the descending colon. The endoscope could not pass through. (**B**) The tumor with mucosal erythema was located at the SD Junction. The endoscope was able to pass through. (**C**) The tumor was located in the sigmoid colon, and the mucosa was red and prone to bleeding, but the endoscope was able to pass through. SD, Sigmoid-Descending colon; SMT, submucosal tumor

**Fig. 2:**
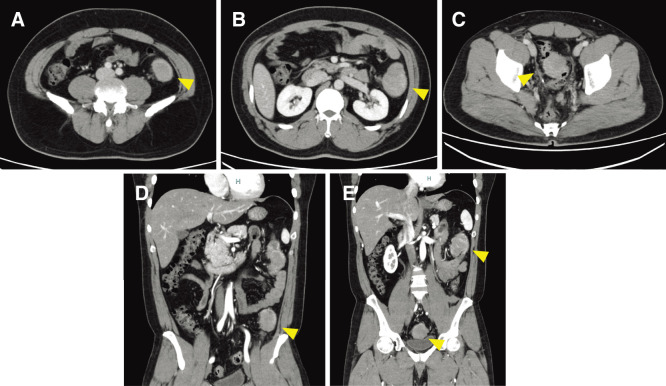
An abdominal contrast CT scan. Three tumors are detected in the descending colon and sigmoid colon. (**A–C**) Axial views. (**D**, **E**) coronal views. Arrowhead: a lead point. (**A**, **D**) The tumor was located at the SD Junction. (**B–D)** The tumors were located in the descending colon and sigmoid colon. CT, computed tomography; SD, Sigmoid-Descending colon

A colonoscopy revealed tumors in the descending colon and sigmoid colon, preventing the passage of the endoscope (**Figs. 1A–1C**). Biopsy results revealed poorly differentiated adenocarcinoma. Chest and abdominal enhanced computed tomography (CT) revealed 3 tumors from the descending colon to the sigmoid colon (**Figs. 2A–2E**). No lymph node metastasis was found, but numerous peritoneal disseminations were found. No lung metastasis was found.

**Fig. 3:**
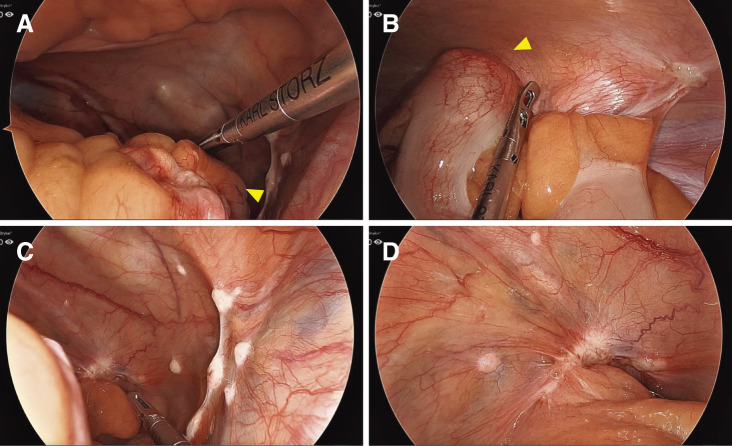
Intraoperative findings. (**A**) The tumor is exposed to the serosal surface and located in splenic flexure (arrowhead, a lead point). (**B**) Tumor located in SD junction (arrowhead, a lead point). (**C**, **D**) Peritoneal dissemination was found in the Douglas’ pouch. SD, Sigmoid-Descending colon

Based on these findings, we diagnosed multiple colon cancers (descending and sigmoid colon cancer) and performed a laparoscopic left hemicolectomy to control the bleeding because of hematochezia. The intraoperative finding was that tumors were in splenic flexure, Sigmoid-Descending colon (SD) junction, and rectosigmoid (**Figs. 3A and 3B**). The tumor in the SD junction invaded the abdominal wall, and peritoneal dissemination was found in the Douglas’ pouch (**Figs. 3C and 3D**). Peritoneal dissemination was not resected. Ascites and liver metastasis were not observed. Reconstruction was selected for double stapling technique (DST) anastomosis using CDH29 (Ethicon, Tokyo, Japan), and colostomy was not created.

**Fig. 4:**
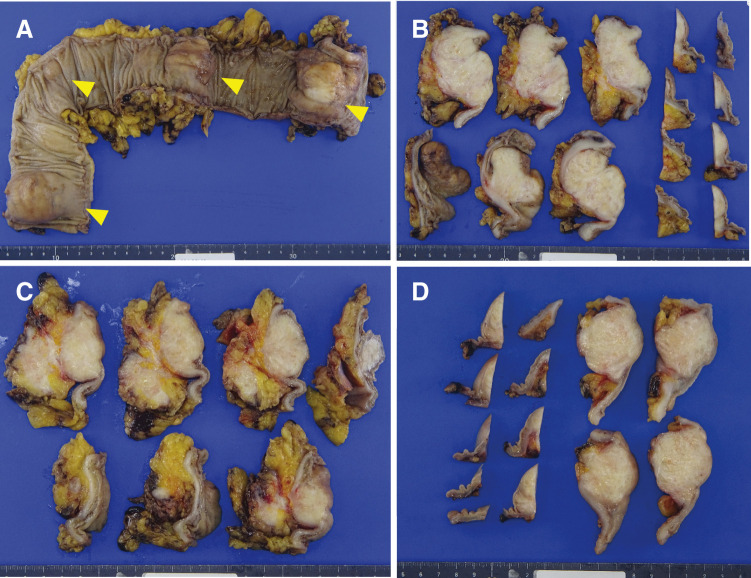
Resected specimen. (**A**) Resected specimen revealed 4 SMT-like tumors. Arrowhead: a lead point. (**B–D**) The cut surfaces of all tumors revealed white solid masses. (**B**, sigmoid colon; **C**, **D**, descending colon.) SMT, submucosal tumor

**Fig. 5:**
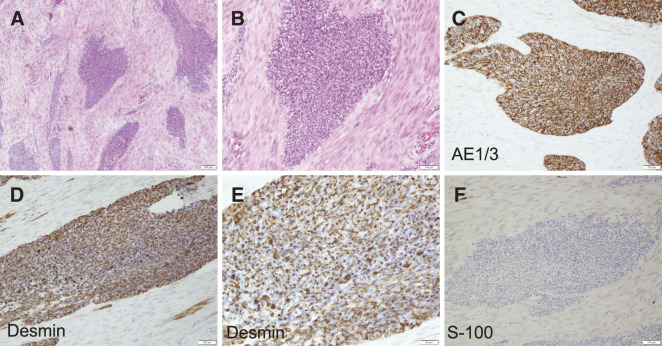
H&E staining and immunohistochemistry findings of excised specimen. (**A**) H&E staining (×50), (**B)** H&E staining (×100), (**C**) AE1/3 (×100), (**D**) Desmin (×100), (**E**) Desmin (×200), and (**F**) S-100 (×100). AE1/3, Cytokeratin-multi (AE1/3); H&E, Hematoxylin–eosin

An excised specimen revealed that 4 tumors were found in the resected intestine. All tumors were submucosal tumor (SMT)-like mucosal protrusions, and the cut surfaces of all tumors revealed white solid masses (**Figs. 4A–4D**). Hematoxylin**–**eosin (H&E) staining showed that in all tumors, atypical cells with large and small swollen nuclei formed irregular solid nests of various sizes against a background of extensive desmoplastic or myxomatous stroma (**Figs. 5A and 5B**). No lymph node metastasis was detected.

Immunohistochemistry showed that tumor cells were CAM5.2 (+), AE1/3 (+), Ber-EP4 (–), EMA (+), CD45 (+), NSE (+), Synaptophysin (–), Chromogranin A (–), CD56 (+), NKX2.2 (–), Vimentin (+), GFAP (–), S-100 (–), Desmin (–), SMA (–), WT1 (–), MyoD1 (–), Myogenin (–) (**Figs. 5C–5F**). Genetic analysis detected the EWS-WT1 fusion gene at another inspection agency. Based on these pathological results, we diagnosed DSRCT.

The patient was discharged on the 19th postoperative day without postoperative complications. Because the case was unresectable with peritoneal dissemination, we decided it was necessary to undergo multidisciplinary treatment, including chemotherapy, and treatment was continued at another hospital.

## DISCUSSION

DSRCT is an aggressive soft-tissue sarcoma as a specific disease first described by Gerard and Rosai in 1989 and officially named in 1991.^5–7)^

Generally, DSRCT occurs in the peritoneal cavity, retroperitoneum, and pelvis, but 6% of DSRCT can occur in an extra-abdominal location and can also be found in the lungs, eyes, and salivary glands.^10–12)^ The origin of DSRCT is unclear. Because most patients develop DSRCT within cavities lined with mesothelial cells and because tumor cells show positive immunohistochemical reactions for epithelial and mesenchymal antigens, such as Desmin and WT1, DSRCT is speculated to originate from mesothelial or submesothelial cells.^13)^ The incidence of DSRCT does not differ significantly between races, but it is predominantly male, with a male-to-female ratio of approximately 3–5:1, and it tends to occur more frequently in younger people.^14)^ DSRCT is characterized by a poor prognosis with 15% overall survival at 5 years.^15)^

DSRCT does not have specific clinical symptoms. Most patients present with early symptoms such as abdominal pain, constipation, ascites, and vomiting.^2–4,14,16)^ Due to the serosal spread of DSRCT, Hayes Jordan, and colleagues at MD Anderson Cancer Center in Houston,^4,14)^ Texas established a new staging system:

Stage 1: Patients with limited disease, localized to 1 or 2 sites in the abdomen or 1 site elsewhere.
Stage 2: Patients with any amount of extensive peritoneal disease.
Stage 3: Patients with liver metastasis and peritoneal disease.
Stage 4: Including peritoneal and liver disease as well as disease outside the abdominal cavity and lymph nodes.

However, this proposed staging system has not yet been validated. DSRCT is very difficult to diagnose preoperatively. Tumor markers such as carbohydrate antigen 125 (CA125) and Neuron-specific enolase (NSE) may be elevated, but there are currently no specific tumor markers.^17)^

Although DSRCT has no special imaging characteristics, CT is the most useful initial imaging test, and magnetic resonance imaging (MRI) is useful in diagnosing pelvic and hepatic lesions. Abdominal CT scans often reveal large, heterogeneous tumors in the abdominal cavity and pelvic peritoneum. Ultrasound often reveals low signal intensity. MRI often shows high signal intensity on T2 weighted image (T2WI) and iso-signal intensity on T1WI^.18,19)^ Also, positron emission tomography (PET)-CT may monitor residual disease and provide early detection of recurrence or tumor progression.^20)^

Histological examination and immunohistochemical staining are essential for differential diagnosis. The differential diagnosis of DSRCT can be with a variety of other round-cell tumors, including Ewing sarcoma, rhabdomyosarcoma, small-cell carcinoma, and mesothelioma.^21,22)^ In most cases of DSRCT, the expression of Desmin, Cytokeratin (CK), Epithelial membrane antigen (EMA), and vimentin is positive. Positivity of both Desmin and CK is considered a specific immunodiagnostic indicator of DSRCT. Vimentin positivity suggests that the tumor is derived from myofibroblasts. Such tumors may express epithelial, mesenchymal, neuroendocrine, and other immunophenotypes and have specific cytogenetic profiles.^23,24)^ DSRCT is distinguished by the t (11;22) (p13;q12) chromosomal translocation involving the fusion of the transcriptional activation domain of Ewing’s sarcoma breakpoint region1 (EWSR1) with the WT1 gene.^2)^ Studies have also suggested that the EWSR1-WT1 fusion protein can induce the expression of platelet-derived growth factor A, leading to fibroblast growth and proliferation and the production of a collagenous stroma, which may explain the characteristic reactive fibrosis of DSRCT.^25)^ The EWSR1-WT1 gene fusion forms a chimeric protein acting as a transcription factor with at least 35 known target genes, including platelet-derived growth factor (PDGF),^25)^ insulin-like growth factor-1 (IGF-1) receptor, epidermal growth factor receptor (EGFR), and others such as Myc proto-oncogene protein (c-MYC) and fibroblast growth factor receptor (FGFR). This translocation and the resulting transcriptional changes are believed to be the major drivers in DSRCT^.26)^ Investigation of somatic mutations, copy number alterations (CNA), and chromosomes in DSRCT samples suggested that deregulation of mesenchymal–epithelial reverse transition (MErT)/epithelial–mesenchymal transition (EMT) and DNA damage repair (DDR) may be important in DSRCT.^27)^

There is no established optimal treatment for DSRCT. Currently, multimodal therapy combining chemotherapy, aggressive cytoreductive surgery, adjuvant radiotherapy, and hyperthermic intraperitoneal chemotherapy (HIPEC) is considered the standard of care for patients without extraperitoneal metastases.^28–30)^ As most cases present as intraperitoneal tumors, the main guidelines recommend initiating treatment with systemic chemotherapy.

Surgery for DSRCT has been reported to be beneficial. Hassan et al. reported that patients who underwent surgical resection had a median survival of 34 months compared with 14 months for non-resected patients.^31)^ Wong et al. reported that patients who underwent abdominal or pelvic tumor resection had a median survival of 47 months compared with 16 months for non-resected patients.^32)^

The most effective chemotherapy regimens with curative intent are still debated, but most are based on regimens used to treat other small round cell sarcomas, combining anthracyclines, alkylating agents, and vinca alkaloids. Kushner et al. recommended the P6 protocol (cyclophosphamide 2100 mg/m^2^ (days 1 and 2), doxorubicin 75 mg/m^2^ (days 1 and 2), and vincristine 0.67 mg/m^2^ (day 1) for courses 1, 2, 3, and 6, and ifosfamide 1800 mg/m^2^ (days 1–5) and etoposide 100 mg/m^2^ (days 1–5) for courses 4, 5, and 7), which, when combined with surgical treatment, resulted in a favorable 5-year survival rate of 35%.^33)^

HIPEC has been reported to be effective in cases with good response after chemotherapy and without extraperitoneal metastasis.^31)^ In the Phase II clinical trial of HIPEC for DSRCT following cytoreductive surgery (CRS), reported by Hayes-Jordan et al. in 2018, improvements were observed in both the 3-year overall survival rate and the 3-year recurrence-free survival rate.^30)^ However, some reports have not demonstrated the efficacy of HIPEC after CRS.^34,35)^ Therefore, the benefits of HIPEC are still unclear and need to be evaluated in prospective trials.

Cases of intestinal DSRCT reported previously in the English literature to date are summarized in **Table 2**. The 6 patients, including this case, were 3 males and 3 females, and their ages at diagnosis ranged from 14 to 56 years. DSRCT has a poor prognosis and requires multimodal therapy, but 3 patients did not wish to undergo it. All 3 patients who did not wish to undergo multimodal therapy have died.

**Table 2:** Clinical summary of intestinal DSRCT

Authors	Age/Sex	Surgery	Metastasis before operation	Additional therapy after operation	Recurrence (Yes/No)	Recurrence site	Time to recurrence (months)	Alive or dead	Time to death (months)
Chen et al.^1)^	18/M	Exploratory laparotomy	Peritoneum, liver, pleura, bone, muscle	ND				Dead	NS
Laurens et al.^43)^	56/F	En-block excision of tumor	Peritoneum	ND	No			NS	
Pahuja et al.^44)^	NS/M	En-block excision of tumor	Peritoneum	P6 regimen and RT	No			Dead	22
Huang et al.^45)^	30/F	Transverse colectomy	No	ND	No			NS	
Liu et al.^46)^	14/F	PD	Lymph nodes	Carboplatin and duoxitasai	Yes	Intraperitoneal	2	Dead	6
Present case	38/M	Laparoscopic left hemicolectomy	Peritoneum					Alive	

DSRCT, desmoplastic small round cell tumor; ND: not done, NS: not specified, PD: pancreaticoduodenectomy, RT: radiotherapy

Current research is focused on developing targeted immunotherapy using monoclonal antibodies directed at DSRCT antigens.^36)^ Two DSRCT cell surface antigens have been identified: GD2, recognized by the antibody 3F8, and the immunomodulatory molecule B7H3, recognized by the antibody 8H9.^37,38)^ Other clinical trials are exploring experimental treatments such as the receptor tyrosine kinase (RTK) inhibitor Pazopanib, dopamine-like receptor 2 antagonist ONC201, intraperitoneal radioimmunotherapy with 131I-omburtamab, and HIPEC^.30,39–41)^ Lamhamedi-Cherradi et al. employed enzalutamide and androgen receptor-directed antisense oligonucleotides (AR-ASO) to inhibit DSRCT cell growth induced by 5α-dihydrotestosterone, significantly decreasing xenograft tumor burden and highlighting the efficacy of androgen-targeted therapies.^42)^

Currently, potential therapeutic targets for DSRCT are under development.^36)^

## CONCLUSIONS

We experienced a very rare case of DSRCT. DSRCT is a fatal disease that primarily affects adolescent and young adult males. Currently, there is no proven treatment. More case reports are essential to improve the management of this disease.

## DECLARATIONS

### Funding

No funding was received for this study.

### Authors’ contributions

All authors managed the perioperative course.

NT, MT, KT, and YY performed the operation.

NT contributed to the drafting and the manuscript.

MT, DU, KI, SM, SM, TY, KN, YY, MN, KT, TH, DT, HY, MT, and YA contributed to the revision of the manuscript critically for important intellectual content and have made final approval of the manuscript.

All authors have read and approved the manuscript, and they are responsible for the manuscript.

### Availability of data and materials

The data that support the findings of this study are available from the corresponding author upon reasonable request.

### Ethical approval and consent to participate

This work does not require ethical considerations or approval.

### Consent for publication

Consent was obtained from the patient to publish the clinical and imaging data in this report.

### Competing interests

The authors declare that they have no competing interests.
